# Non-destructive assessment of soluble solids content in kiwifruit using hyperspectral imaging coupled with feature engineering

**DOI:** 10.3389/fpls.2024.1292365

**Published:** 2024-01-31

**Authors:** Wei Xu, Liangzhuang Wei, Wei Cheng, Xiangwei Yi, Yandan Lin

**Affiliations:** ^1^ Institute for Electric Light Sources, School of Information Science and Technology, Fudan University, Shanghai, China; ^2^ Institute for Six-sector Economy, Fudan University, Shanghai, China; ^3^ Academy for Engineering & Technology, Fudan University, Shanghai, China

**Keywords:** kiwifruit, soluble solids content, feature engineering, stacking generalization, spectral imaging

## Abstract

The maturity of kiwifruit is widely gauged by its soluble solids content (SSC), with accurate assessment being essential to guarantee the fruit’s quality. Hyperspectral imaging offers a non-destructive alternative to traditional destructive methods for SSC evaluation, though its efficacy is often hindered by the redundancy and external disturbances of spectral images. This study aims to enhance the accuracy of SSC predictions by employing feature engineering to meticulously select optimal spectral features and mitigate disturbance effects. We conducted a comprehensive investigation of four spectral pre-processing and nine spectral feature selection methods, as components of feature engineering, to determine their influence on the performance of a linear regression model based on ordinary least squares (OLS). Additionally, the stacking generalization technique was employed to amalgamate the strengths of the two most effective models derived from feature engineering. Our findings demonstrate a considerable improvement in SSC prediction accuracy post feature engineering. The most effective model, when considering both feature engineering and stacking generalization, achieved an $RMSE_{p}$ of 0.721, a $MAPE_{p}$ of 0.046, and an $RPD_{p}$ of 1.394 in the prediction set. The study confirms that feature engineering, especially the careful selection of spectral features, and the stacking generalization technique are instrumental in bolstering SSC prediction in kiwifruit. This advancement enhances the application of hyperspectral imaging for quality assessment, offering benefits that extend across the agricultural industry.

## 1 Introduction

Kiwifruit (Actinidia deliciosa) is a popular fruit known for its unique flavor and nutritional benefits. As a typical climacteric fruit, it continues ripening even after being harvested. This post-harvest ripening process makes kiwifruit highly perishable and requires careful handling and storage to maintain its quality. The assessment of its quality and maturity commonly relies on the measurement of soluble solids content (SSC). On the one hand, SSC serves as an indicator of the sugar content in kiwifruit, for sugars constituting approximately 81% of the total SSC (Tian et al., 2022). On the other hand, SSC exhibits a consistent pattern of variation over time in storage. Throughout the storage period, as time goes by, the starch and pectin present in the kiwifruit undergo hydrolysis, leading to a gradual increase in SSC. Therefore, monitoring the SSC of kiwifruit is effective for evaluating its quality and maturity. However, the determination of SSC, being an internal attribute of fruit, often involves destructive techniques like refractometry, which requires the extraction of juice or pulp from fruit. These methods are time-consuming, labor-intensive and cause damage to the fruit, preventing the repeated utilization of samples. Consequently, there is an increasing demand for non-destructive and expeditious techniques that can precisely estimate the SSC of kiwifruit.

Hyperspectral imaging has emerged as a promising non-destructive method for assessing the quality of various agricultural products (Yao et al., 2013; Huang et al., 2018). This technique enables the measurement of spectral reflectance across a broad range of wavelengths, providing detailed insights into the chemical and physical properties of samples. In the case of kiwifruit, the visible near-infrared (Vis-NIR) spectral range contains valuable information related to the absorption of O–H, N–H, and C–H vibrations (Guo et al., 2017; Xu et al., 2023). These vibrational modes facilitate the identification and quantification of key chemical constituents associated with SSC, such as sugars and other organic compounds. Through the employment of regression models, relevant information can be extracted from spectral reflectance, leading to the establishment of a strong relationship between the observed spectral features and SSC measurements. Once the regression model is constructed, predicting SSC becomes a straightforward process, allowing for the non-destructive estimation of SSC values (Nicolaï et al., 2007).

Various well-designed regression models, such as partial least squares regression (PLSR) (Lee et al., 2022), support vector machine regression (SVR) (Ma et al., 2018), and artificial neural network (ANN) (Pullanagari and Li, 2021) have been developed to establish the relationship between observed spectral features and SSC measurements. However, the high-dimensional nature of spectral features can pose challenges to regression models. These features often contain redundant information and are influenced by various disturbances (e.g., sample differences, environmental noise, and baseline drift). Excessive redundant information for regression models not only results in prolonged hardware and software runtime but also compromises the regression performance, leading to unreliable estimations of SSC values (Xiaobo et al., 2010).

Unlike previous research that focuses on refining regression or machine learning models, our study intentionally emphasizes the importance of eliminating redundancies and disturbances in the initial phase of model development to enhance SSC prediction for kiwifruit—a crucial yet frequently underestimated step in existing studies.

The quality and suitability of input features significantly influence the performance of regression models. Carefully selected features provide more relevant information, resulting in simpler models and improved results. Conversely, the inclusion of irrelevant features can negatively impact the model’s ability to generalize. In contrast to complex models, which may present challenges in interpretation and fine-tuning, simpler models with more effective features tend to yield more reliable results (Xiaobo et al., 2010). Hence, it is essential to pay meticulous attention to the pre-processing and selection of these features. These tasks, involving data converting and filtering before model building, are collectively referred to as feature engineering. In general, feature engineering involves spectral pre-processing and selection to effectively mitigate the impact of various disturbances, eliminate irrelevant features, and identify the most informative ones. Its ultimate goal is to generate enhanced features that are well-suited for integration into regression models. By prioritizing the use of more effective features and employing simple models, we can strike a balance between model complexity and performance, thus leading to more accurate and interpretable regression results.

In this study, we focus on investigating the effectiveness of feature engineering in enhancing the performance of SSC prediction in kiwifruit using hyperspectral imaging. To achieve this goal, we employed a linear regression model based on ordinary least squares (OLS) due to its simplicity and interpretability. Subsequently, we conducted a systematic evaluation and comparison of the variations in the regression performance under different combinations of four spectral pre-processing methods and nine spectral feature selection methods (details will be provided in section 2.3~2.5). Through this comprehensive analysis, our study not only demonstrates the positive impact of feature engineering but also identifies the optimal condition that yields the best regression performance. Additionally, we introduce the stacking generalization technique to integrate the strengths of two best-performing models which are achieved through above feature engineering, thus effectively addressing overfitting issues, and further improving the regression performance. This study highlights the potential of feature engineering and the stacking generalization technique in SSC prediction for kiwifruit, providing practical insights for quality assessment in the kiwifruit industry. The application of these techniques holds promise for more efficient and reliable SSC prediction, benefiting the kiwifruit industry and potentially extending to other agricultural produce quality assessment domains.

## 2 Materials and methods

### 2.1 Preparation of kiwifruit samples

In June 2023, a substantial number of kiwifruit samples were obtained from an agricultural plantation situated in Shaanxi Province, China. Following the removal of unqualified samples such as unripe, overripe, or mechanically damaged ones, a total of 116 kiwifruit samples with intact skin were selected for utilization in this experiment.

Prior to conducting the spectral acquisition step, a meticulous wiping procedure was carried out using soft tissue paper to eliminate any lint present on the surface of kiwifruit samples. This step was taken to mitigate the potential influence of lint on the spectral acquisition step.

Immediately following the spectral acquisition step, the sample preparation for the SSC measurement was conducted under the guidelines of the NY/T 2637-2014 standard. This sample preparation entails peeling the samples along their equators, removing the pulp, and extracting the juice through pressing. The kiwifruit juice will be introduced into the detection tank of one refractometer for subsequent SSC measurement.

### 2.2 Spectral acquisition and SSC measurement

**Figure 1:**
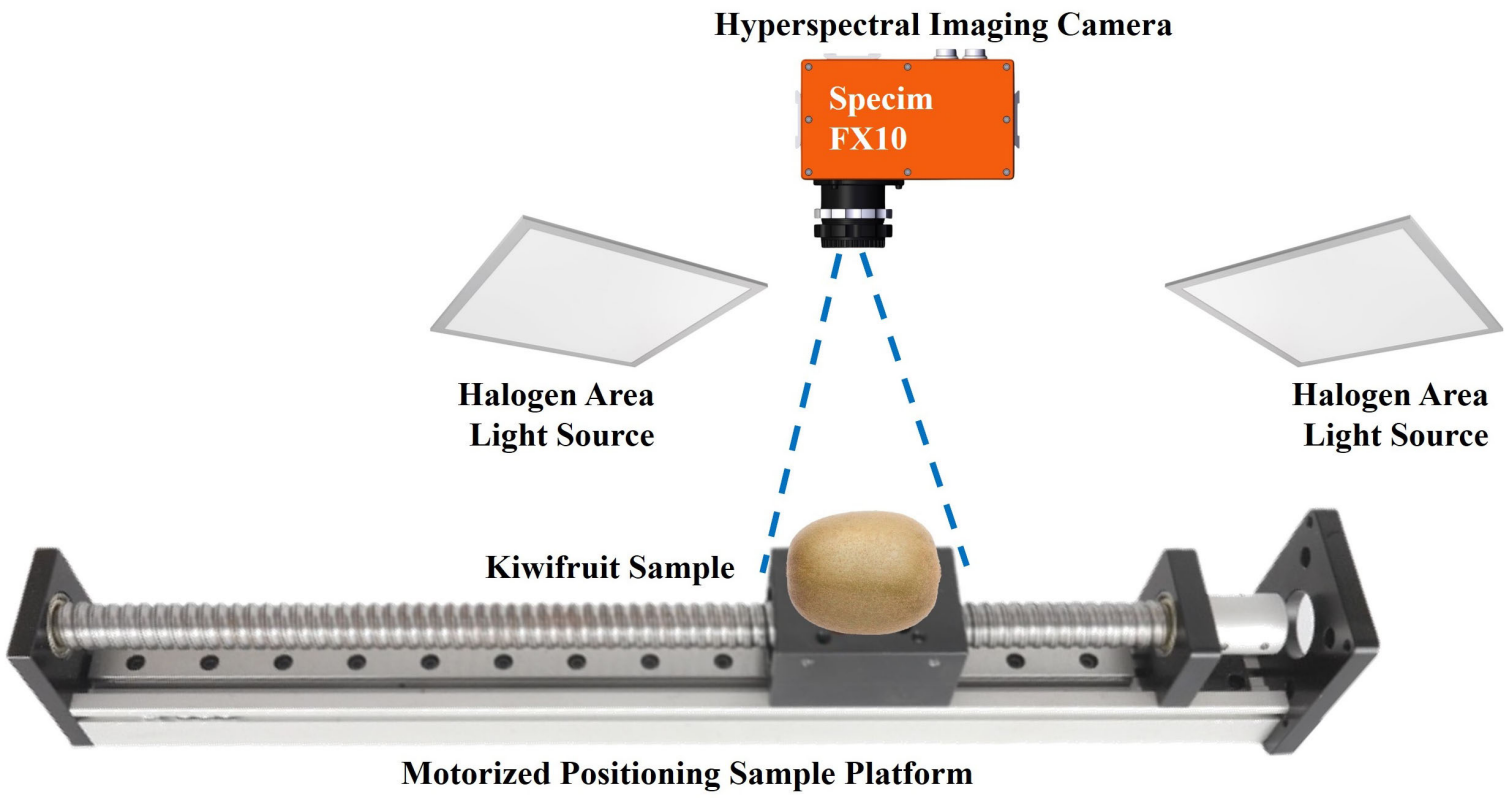
The custom-built hyperspectral imaging system.

A custom-built hyperspectral imaging system is specifically developed to capture spectral images of the kiwifruit samples, consisting of four main components: a spectral imaging camera (Specim FX10, Konica Minolta, Inc., Japan), a motorized positioning sample platform, two halogen area light sources, and a computer installed with suitable data acquisition software (see **Figure 1**). Among them, the Specim FX10 spectral imaging camera provides a spectral resolution of 400 ~ 1000 nm (due to the low signal-to-noise ratio in the lower wavelength regions, only data from wavelengths above 450 nm were exclusively utilized in this study) and works in a push-broom mode, thus necessitating a motorized positioning sample platform. To ensure an accurate aspect ratio in the captured spectral images, it is crucial to carefully adjust the advancing speed of the platform and the exposure time of the spectral imaging camera to match each other. The two light sources were positioned symmetrically to uniformly illuminate the camera’s field of view. This arrangement aims to ensure consistent spectral response across different positions within the imaged area. For stable and accurate measurements, a one-hour warm-up and black and white calibration procedure should be performed before the initial use of the system. Besides, the whole procedure of spectral acquisition was performed in a dark room to avoid the interference of stray light.

A digital refractometer with a resolution of 0.1% Brix (PAL-1, ATAGO Inc., Japan) was utilized to measure the SSC of kiwifruit samples. First, the prepared kiwifruit juice was carefully dropped into the detection tank. Then, the SSC physicochemical values of SSC were recorded once the display data stabilized. It is worth noting that before measuring the SSC of each sample, it is essential to calibrate the refractometer reading by setting it to zero using distilled water. This calibration step was crucial to ensure the accuracy and reliability of the SSC measurements by accounting for any potential offset or drift in the refractometer readings.

### 2.3 Feature engineering

Feature engineering involves two key aspects: spectral pre-processing methods and spectral feature selection methods. Spectral pre-processing refines spectral reflectance by mitigating disturbances, while spectral feature selection eliminates redundancy, pinpointing crucial informative attributes for modeling. This duality is essential for extracting meaningful patterns from raw data and is imperative for developing robust regression models.

Recognizing that feature quality significantly influences model success, we implement an orthogonal experimental design for feature engineering. This methodical approach ensures experimentation and validation tailored to our specific modeling context, enabling a structured assessment of diverse feature engineering strategies’ effects on model accuracy. We rigorously investigate four spectral pre-processing and nine spectral feature selection techniques, assessing their individual and combined effects. The ensuing sections, 2.4 and 2.5, will delineate these techniques, underscoring their roles in data refinement and feature optimization, ultimately contributing to the improved accuracy of our model.

### 2.4 Spectral pre-processing methods

During the spectral acquisition step, various disturbances, such as sample differences, environmental noise, and baseline drift, can affect the final captured spectral image (Xu et al., 2023). To mitigate these variations in spectral reflectance and emphasize the features related to SSC, a spectral pre-processing procedure is conducted. It is a critical step in feature engineering (Lee et al., 2022) and primarily aims to refine and cleanse the data by removing unwanted noise, correcting baseline drift, and addressing other disturbances. To tackle the specific variations encountered in spectral pre-processing, a wide array of algorithms has been developed, each possessing unique characteristics and catering to various aspects of the process. In the following content, we will provide a brief description of several widely used spectral pre-processing methods that will be utilized in this study later.

Firstly, the Standard Normal Variant transform (SNV) (Dong et al., 2022; Liu et al., 2022) is a notable method that is meticulously designed to address the detrimental effects of scattering and concentration-related influences. It achieves this by normalizing spectral reflectance across the entire wavelength range, effectively mitigating deviations, and nullifying the impact of extraneous factors. Secondly, the Direct Orthogonal Signal Correction (DOSC) (Westerhuis et al., 2001) method disentangles spectral reflectance into correlated and uncorrelated components. By leveraging the principles of multivariate statistics, it discriminates between valuable signal information and intrusive background perturbations. In addition, the Detrend Correction (DC) (Ai et al., 2022) method adeptly attenuates the disruptive interference of external noise. It accomplishes this by subtracting the trend-fitting lines, enabling a refined and noise-free characterization of intrinsic spectral attributes. Lastly, the Savitzky-Golay (SG) (Savitzky and Golay, 1964) convolution smoothing method emerges as an exemplary technique for spectral refinement. By utilizing weighted polynomial regression within moving windows, it effectively suppresses high-frequency noise while preserving essential spectral features.

### 2.5 Spectral feature selection methods

Spectroscopy instruments typically exhibit highly correlated spectral responses, particularly in adjacent wavelength regions, leading to redundant data. Additionally, not all wavelengths are relevant to the problem at hand, potentially impacting the accuracy and precision of results. Therefore, discriminative feature selection becomes critical to enhance model performance. A range of spectral feature selection methods was investigated to address these issues, which are integral to feature engineering. These methods aim to identify and retain informative features, reduce the feature space, improve computational efficiency, and prevent multicollinearity and overfitting. Nine distinct spectral feature selection methods were identified and classified into three categories: basis-vectors-based, statistical-measures-based, and iterations-based methods. Each category offers unique approaches to feature selection and is briefly described below.

#### 2.5.1 Based on basis vectors

Dimensionality reduction techniques such as Principal Component Analysis (PCA) (Pearson, 1901; Hotelling, 1933) and Singular Value Decomposition (SVD) (Smithies, 1938) use linear combinations of basis vectors to simplify high-dimensional data. PCA prioritizes components based on explained variance, while SVD utilizes singular values. Additionally, Kernel Principal Component Analysis (KPCA) (Schölkopf et al., 1997) extends PCA by capturing nonlinear patterns through a higher-dimensional kernel-based feature space, providing greater flexibility in representing high-dimensional data and extracting nonlinear features. By selecting a subset of basis vectors and transforming, these dimensionality reduction methods effectively reduce the dimensionality of the data while endeavoring to preserve as much information as possible.

#### 2.5.2 Based on statistical measures

Individual wavelength features can also be evaluated using statistical measures. The F-test assesses the significance of feature differences between classes. Features with high F-values indicate greater relevance. Thus, one can rank the features based on their F-values and select the top n features for further analysis or dimensionality reduction. Similarly, the Pearson product-moment correlation coefficient (PPMCC) measures linear correlations, while Mutual Information (MI) detects both linear and non-linear dependencies.

#### 2.5.3 Based on iterations

Iterative feature selection methods systematically search the feature space to identify the most relevant features for a specific problem. These methods, through a process of selection and elimination, adaptively integrate criteria, performance metrics, or domain knowledge. The Recursive Feature Elimination (RFE) (Araújo et al., 2001) is one such method that employs a backward elimination technique to prune irrelevant features from a regression model. Starting with all features, RFE trains the model, ranks features by their impact on model performance, and iteratively discards the weakest until a targeted feature set size or stopping condition is reached. The Successive Projection Algorithm (SPA) (Soares et al., 2013) selects features by projecting data onto orthogonal hyperplanes, treating spectral feature selection as a constrained combinatorial optimization problem. SPA minimizes multicollinearity, thereby reducing redundancy and addressing ill-conditioning by preventing the propagation of superfluous features during calibration. The Competitive Adaptive Reweighted Sampling (CARS) (Li et al., 2009; Zhang et al., 2019) focuses on discarding features with minor regression coefficients in the PLSR model, using adaptive reweighting and cross-validation to fine-tune feature selection. CARS’ adaptability allows it to dynamically capture dataset characteristics, which may result in varying feature selections across iterations.

### 2.6 Experiment settings

#### 2.6.1 Sample division

The Sample Set Partitioning Based on Joint X-Y Distances (SPXY) (Wang et al., 2022) method was employed to divide the entire dataset of 116 kiwifruit samples into a calibration set and a prediction set, with a ratio of 3:1. This hold-out partitioning technique ensures a representative distribution of samples across both sets, allowing for the evaluation of model performance on unseen data.

Furthermore, the number of selected features in the spectral feature selection methods was determined using 5-fold cross-validation on the calibration set. This approach optimizes the feature selection process by iteratively evaluating the performance of different feature subsets across various subsets of the calibration set. By employing cross-validation, the optimal number of selected features is achieved while mitigating the risk of overfitting and ensuring the robustness of the model’s performance.

#### 2.6.2 Evaluation metrics

**Figure 2:**
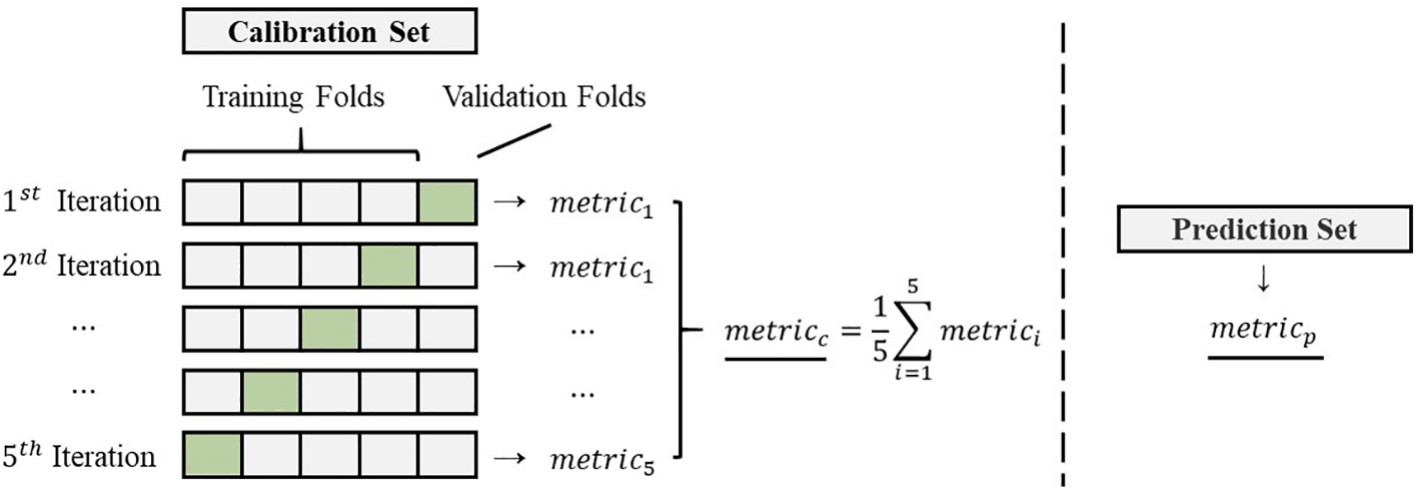
The details of the sample division and metrics calculation.

Three metrics, namely the Root Mean Square Error (RMSE), the Mean Absolute Percentage Error (MAPE), and the Residual Prediction Deviation (RPD) were employed to evaluate the impact of feature engineering on the regression model. These evaluation metrics are calculated using the following Equations 1–3.


(1) $$\begin{array}{c} RMSE=\sqrt{\frac{1}{N}\sum_{i=1}^{n}{\left(y_{i}-{\hat{y}}_{i}\right)}^{2}} \end{array}$$

(2) $$\begin{array}{c} MAPE=\frac{1}{N}\sum_{i=1}^{n}\frac{\left|y_{i}-{\hat{y}}_{i}\right|}{y_{i}} \end{array}$$

(3) $$\begin{array}{c} RPD=\frac{SD}{RMSE} \end{array}$$

where ${\hat{y}}_{i}$ is the predicted value of the $i^{th}$ sample, $y_{i}$ is the measured value of the $i^{th}$ sample, and N is the total number of samples in the prediction set. Additionally, SD is the standard deviation of the measured value of the N samples. It is important to note that the metrics calculated for the validation set ( $RMSE_{v}$ , $MAPE_{v}$ and $RPD_{v}$ ) represent the mean values obtained from cross-validation. Conversely, the metrics calculated for the prediction set ( $RMSE_{p}$ , $MAPE_{p}$ and $RPD_{p}$ ) represent the mean values obtained from a single prediction. The details of the sample division and metrics calculation can be found in **Figure 2**.

#### 2.6.3 The regression model

To comprehensively evaluate the effectiveness of feature engineering, a linear regression model based on OLS was established using an orthogonal experimental design. The OLS model, known for its ability to minimize the sum of squared residuals, is a widely-used regression method and a suitable choice for modeling the relationship between the input features and the SSC values. Its simplicity and interpretability make it a solid foundation for analyzing and comparing the effects of feature engineering on the regression model’s performance. Meanwhile, those orthogonal experiments allow for a thorough examination of the individual effects of spectral pre-processing methods and spectral feature selection methods, as well as the exploration of potential interactions between them. By systematically varying and controlling these factors, researchers can gain valuable insights into the impact of different feature engineering techniques on the overall performance of the regression model.

## 3 Results and discussion

### 3.1 Distribution of the spectral reflectance and SSC

**Figure 3:**
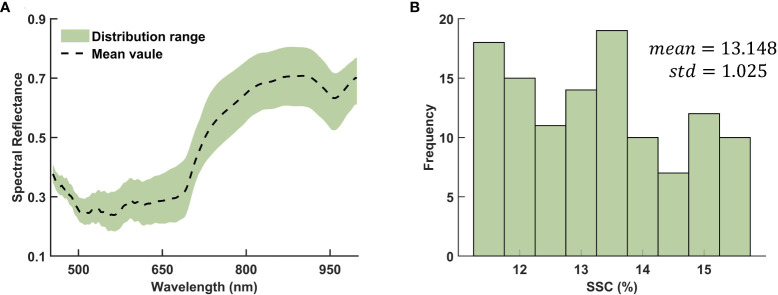
Distribution of the **(A)** spectral reflectance and **(B)** SSC.

The distribution range of spectral reflectance in different wavelength regions were shown in **Figure 3**. Notably, the distribution range below 500 nm appears narrower, indicating lower variance and suggesting that this region contains less information. Conversely, the distribution range above 750 nm is broader, indicating higher variability in spectral reflectance within this wavelength region. This observation suggests that features of higher wavelength regions may contain more valuable information for the analysis and prediction of SSC values.

The SSC values for the complete dataset of 116 kiwifruit samples exhibit a mean value of 13.148 and a standard deviation of 1.025. The distribution of these values approximately follows a normal distribution, as evidenced by the Lilliefors test with a *p*-value of 0.0642. A visual representation of the frequency histogram depicting the specific distribution can be found in **Figure 3**. The calibration set of 87 samples presents a mean SSC of 13.165 and a standard deviation of 1.031, while the prediction set of 29 samples has a mean of 13.093 and a standard deviation of 1.022, indicating similar distribution parameters. Such comparability between calibration and prediction sets is vital to the reliability of our model’s performance evaluation.

### 3.2 Regression performances

The performances of the OLS model under all conditions were summarized in **Tables 1**–**5**, grouped by spectral pre-processing methods, with the best scores highlighted in bold (due to rounding of specific metric values, some values that appear to be the same may still have minor differences). For a clearer comparison of outcomes among different spectral selection methods, we underline the results that fall below the baseline performance (i.e., without employing any spectral selection method) under identical spectral preprocessing conditions. The number of selected features of the corresponding spectral selection method is briefly represented by n .

**Table 1:** Regression performances using various spectral selection methods under no spectral pre-processing.

Pre-processing	Feature Selection	*n*	Calibration (Cross-Validation)			Validation		
			*RMSE_c_*	*MAPE_c_*	*RPD_c_*	*RMSE_p_*	*MAPE_p_*	*RPD_p_*
None	None	/	1.279	0.078	0.811	1.161	0.071	0.865
	PCA	5	0.953	0.062	1.061	0.780	0.053	1.288
	KPCA	5	0.947	0.062	1.068	0.780	0.053	1.287
	SVD	4	0.959	0.063	1.055	0.807	0.055	1.244
	F-test	10	0.966	0.061	1.050	0.943	0.059	1.064
	PPMCC	2	1.014	0.067	0.997	1.021	0.063	0.984
	MI	6	0.937	0.058	1.086	0.883	0.060	1.137
	RFE	6	0.994	0.065	1.017	0.773	0.050	1.299
	SPA	2	0.970	0.063	1.044	0.854	0.055	1.176
	CARS	8	**0.912**	**0.058**	**1.118**	**0.771**	**0.048**	**1.302**

The best scores are highlighted in bold.

**Table 2:** Regression results using various spectral selection methods under SNV spectral pre-processing.

Pre-processing	Feature Selection	*n*	Calibration (Cross-Validation)			Validation		
			*RMSE_c_*	*MAPE_c_*	*RPD_c_*	*RMSE_p_*	*MAPE_p_*	*RPD_p_*
SNV	None	/	1.795	0.109	0.593	1.535	0.098	0.654
	PCA	4	0.958	0.063	1.056	0.840	0.056	1.195
	KPCA	7	0.953	0.063	1.061	**0.740**	**0.048**	**1.358**
	SVD	4	0.958	0.063	1.056	0.840	0.056	1.195
	F-test	10	0.960	0.061	1.058	0.988	0.058	1.016
	PPMCC	2	1.018	0.067	0.995	1.030	0.064	0.975
	MI	4	1.001	0.064	1.019	0.948	0.061	1.059
	RFE	5	0.978	0.065	1.036	0.880	0.058	1.142
	SPA	8	**0.928**	**0.059**	**1.101**	0.795	0.049	1.263
	CARS	8	1.025	0.065	0.999	0.982	0.062	1.023

The best scores are highlighted in bold.

**Table 3:** Regression results using various spectral selection methods under DOSC spectral pre-processing.

Pre-processing	Feature Selection	*n*	Calibration (Cross-Validation)			Validation		
			*RMSE_c_*	*MAPE_c_*	*RPD_c_*	*RMSE_p_*	*MAPE_p_*	*RPD_p_*
DOSC	None	/	/	/	/	/	/	/
	PCA	4	0.974	0.064	1.038	**0.809**	**0.052**	**1.242**
	KPCA	4	0.974	0.064	1.038	0.809	0.052	1.242
	SVD	3	0.984	0.065	1.029	0.845	0.055	1.188
	F-test	5	0.953	0.062	1.061	0.812	0.052	1.236
	PPMCC	5	0.953	0.062	1.061	0.812	0.052	1.236
	MI	2	0.980	0.064	1.033	0.864	0.054	1.162
	RFE	3	1.003	0.066	1.010	0.891	0.058	1.127
	SPA	13	0.970	0.061	1.053	0.879	0.057	1.142
	CARS	12	**0.888**	**0.053**	**1.173**	0.978	0.058	1.026

The best scores are highlighted in bold.

**Table 4:** Regression results using various spectral selection methods under DC spectral pre-processing.

Pre-processing	Feature Selection	*n*	Calibration (Cross-Validation)			Validation		
			*RMSE_c_*	*MAPE_c_*	*RPD_c_*	*RMSE_p_*	*MAPE_p_*	*RPD_p_*
DC	None	/	1.303	0.081	0.785	1.238	0.076	0.811
	PCA	31	0.934	0.058	1.119	0.802	**0.047**	1.252
	KPCA	9	0.957	0.060	1.069	**0.754**	0.051	**1.332**
	SVD	15	0.942	0.059	1.099	0.759	0.049	1.324
	F-test	1	0.964	0.063	1.050	0.831	0.053	1.208
	PPMCC	1	0.964	0.063	1.050	0.831	0.053	1.208
	MI	7	0.982	0.062	1.043	0.810	0.053	1.239
	RFE	5	1.004	0.066	1.011	0.810	0.053	1.240
	SPA	4	0.968	0.064	1.048	0.780	0.049	1.287
	CARS	18	**0.760**	**0.047**	**1.372**	1.189	0.077	0.844

The best scores are highlighted in bold. The results that fall below the baseline performance are highlighted in underlined.

**Table 5:** Regression results using various spectral selection methods under SG spectral pre-processing.

Pre-processing	Feature Selection	*n*	Calibration (Cross-Validation)			Validation		
			*RMSE_c_*	*MAPE_c_*	*RPD_c_*	*RMSE_p_*	*MAPE_p_*	*RPD_p_*
SG	None	/	1.553	0.096	0.662	1.425	0.091	0.704
	PCA	5	0.953	0.062	1.061	0.780	0.053	1.287
	KPCA	5	0.948	0.062	1.068	0.780	0.053	1.287
	SVD	4	0.959	0.063	1.055	0.807	0.055	1.244
	F-test	8	0.989	0.063	1.029	0.924	0.059	1.087
	PPMCC	5	1.015	0.065	1.006	0.903	0.057	1.112
	MI	12	0.980	0.062	1.037	0.793	0.053	1.267
	RFE	6	0.963	0.063	1.049	0.774	0.052	1.298
	SPA	2	0.970	0.063	1.044	0.853	0.055	1.178
	CARS	13	**0.895**	**0.053**	**1.139**	**0.740**	**0.046**	**1.358**

The best scores are highlighted in bold.

These tables provide an exhaustive overview of the evaluation metrics, such as RMSE, MAPE, and RPD, enabling easy comparison and identification of the top-performing models within each feature preprocessing group. As shown in **Tables 1**–**5**, the superior performance of the OLS model utilizing feature engineering becomes evident when comparing it to the model without feature engineering. Within each spectral pre-processing method, employing a spectral feature selection method consistently enhanced performance across all metrics for both the calibration and validation sets (except for the minor anomaly of the $MAPE_{p}$ metric for the DC-CARS-OLS model).

This conclusion, however, does not extend to spectral pre-processing methods. For the sake of simplicity, the performance outcomes of the OLS model under just a few selected spectral feature selection methods are succinctly summarized in **Table 6**. It is apparent that spectral pre-processing methods do not always lead to performance enhancements. Nevertheless, a judicious synergy between spectral pre-processing and feature selection methods may facilitate further amelioration of model performance. It is imperative for scholars to meticulously assess these variations when constructing an optimal feature engineering for their specific application.

**Table 6:** Regression results using various spectral pre-processing methods under no and CARS spectral pre-processing.

Pre-processing	Feature Selection	*n*	Calibration (Cross-Validation)			Validation		
			*RMSE_c_*	*MAPE_c_*	*RPD_c_*	*RMSE_p_*	*MAPE_p_*	*RPD_p_*
None	None	/	**1.279**	**0.078**	**0.811**	**1.161**	**0.071**	**0.865**
SNV		/	1.795	0.109	0.593	1.535	0.098	0.654
DOSC		/	/	/	/	/	/	/
DC		/	1.303	0.081	0.785	1.238	0.076	0.811
SG		/	1.553	0.096	0.662	1.425	0.091	0.704
None	CARS	8	0.912	0.058	1.118	0.771	0.048	1.302
SNV		8	1.025	0.065	0.999	0.982	0.062	1.023
DOSC		12	0.888	0.053	1.173	0.978	0.058	1.026
DC		18	**0.760**	**0.047**	**1.372**	1.189	0.077	0.844
SG		13	0.895	0.053	1.139	**0.740**	**0.046**	**1.358**

The best scores are highlighted in bold. The results that fall below the baseline performance are highlighted in underlined.

These findings underscore the effectiveness of feature engineering in enhancing the regression model’s predictive capabilities. In the calibration set, the DC-CARS-OLS model consistently demonstrates the best performance across all evaluation metrics ( $RMSE_{v}=0.760$ , $MAPE_{v}=0.047$ and $RPD_{v}=1.372$ ), indicating that the combination of the DC spectral preprocessing method, the CARS spectral feature selection method, and the OLS regression model yields the most accurate and reliable predictions in this particular dataset. However, the performance differs in the validation set, where the SG-CARS-OLS model outperforms the other models, achieving the best scores in all evaluation metrics ( $RMSE_{p}=0.740$ , $MAPE_{p}=0.046$ and $RPD_{p}=1.358$ ). This suggests that the combination of the SG spectral preprocessing method, the CARS spectral feature selection method, and the OLS regression model performs exceptionally well on unseen data. These findings emphasize the importance of evaluating model performance in both the calibration set and validation set to ensure the generalizability of the results. It further demonstrates that the optimal combination of feature preprocessing methods and spectral feature selection methods may vary depending on the dataset and the specific task concerned. Researchers should carefully consider these variations when designing the most suitable combination of feature engineering.

The frequency with which the OLS model achieves the best performance for each metric under every condition is summarized in **Table 7**. Among the spectral pre-processing methods, all exhibit an equal frequency of best performance. However, when considering spectral feature selection methods, it is noteworthy that the CARS method stands out with a significantly higher frequency of best performance compared to the other methods. This observation raises the possibility that greater attention should be directed toward spectral feature selection methods during the design of feature engineering and suggests that CARS is particularly effective in selecting informative features for enhancing the performance of the regression model.

**Table 7:** Statistics of the frequency of best performance for each metric under every condition.

	None	PCA	KPCA	SVD	F-test	PPMCC	MI	RFE	SPA	CARS	SUM
None	0	0	0	0	0	0	0	0	0	6	**6**
SNV	0	0	3	0	0	0	0	0	3	0	**6**
DOSC	0	3	0	0	0	0	0	0	0	3	**6**
DC	0	1	2	0	0	0	0	0	0	3	**6**
SG	0	0	0	0	0	0	0	0	0	6	**6**
SUM	0	4	5	0	0	0	0	0	3	**18**	/

The best scores are highlighted in bold.

### 3.3 Selected optimal features

**Figure 4:**
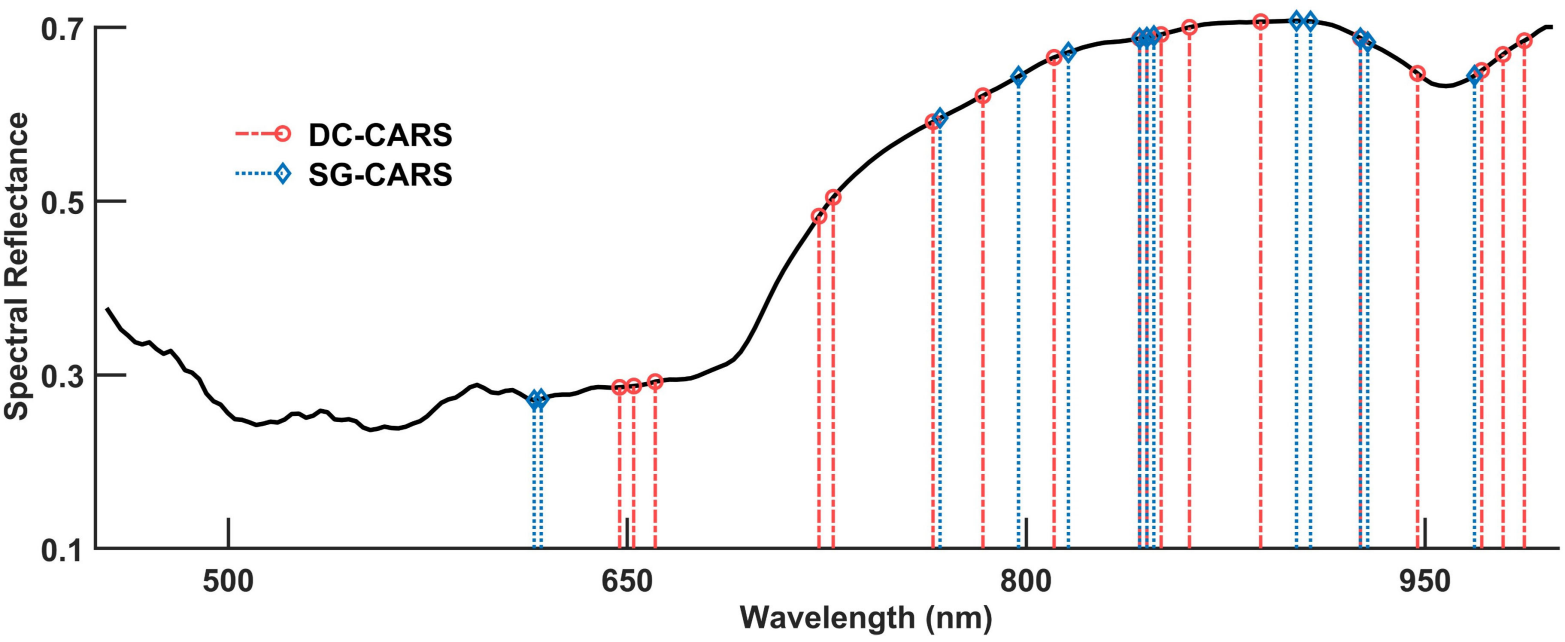
Distribution of the features selected by the DC-CARS and SG-CARS methods.

The distribution of the features selected by the DC-CARS and SG-CARS methods are shown in **Figure 4**. The features extracted by the DC-CARS method show a more dispersed distribution across different wavelengths. In contrast, the features extracted by the SG-CARS method exhibit a relatively concentrated distribution, particularly around 600 nm and 850 nm. Both methods display a concentration of selected features above 750 nm, but there is also a smaller distribution near 600-700 nm. These findings align with the distribution range of spectral reflectance in different wavelength regions, as depicted in **Figure 3A**.

### 3.4 The stacking generalization

We observed that the DC-CARS-OLS model, despite achieving the best performance in the calibration set, did not perform as well in the validation set. This suggests that the DC-CARS-OLS model may have overfit the calibration set and may not generalize well to unseen data. Conversely, the SG-CARS-OLS model achieved the best performance in the validation set but performed lower than the DC-CARS-OLS model in the calibration set, indicating that there is still room for improvement in its fitting ability.

**Figure 5:**
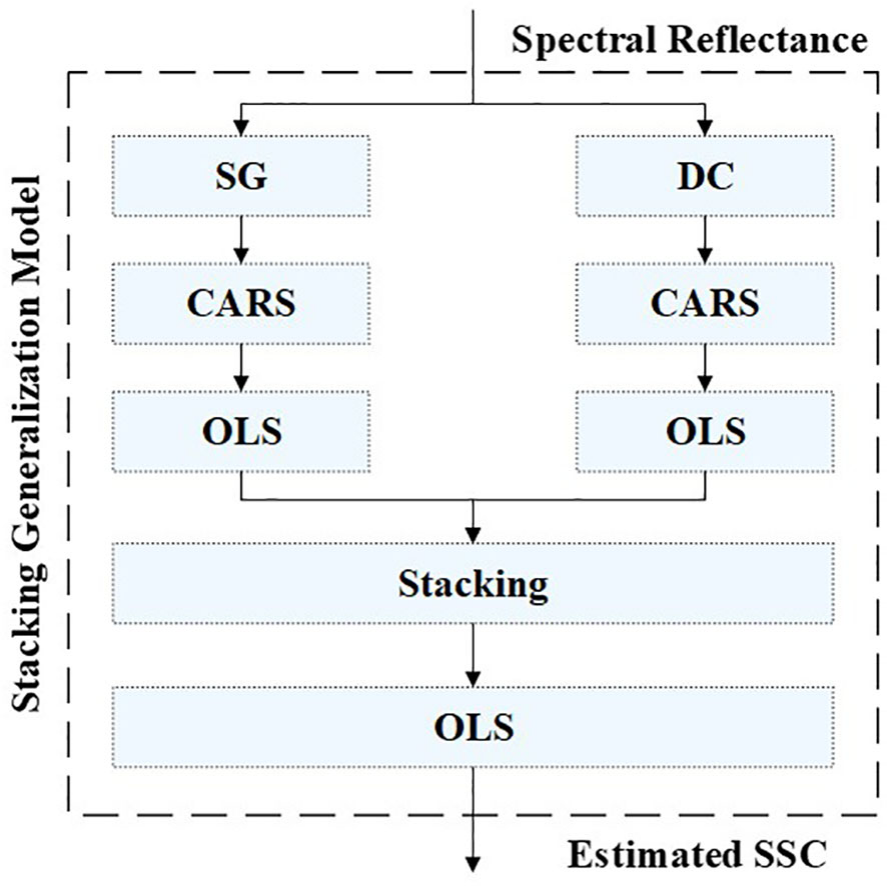
The specific structure and computational flow of the stacking generalization model.

To leverage the strengths of both models and address these limitations, we introduced the stacking generalization technique (Wolpert, 1992). The stacking generalization technique is a powerful method that combines outputs of multiple base models to improve the final predictive performance. It involves constructing a meta-model that takes the predictions of base models as input, thus addressing the limitations of individual models and harnessing their complementary strengths. Specifically, the base models are trained on the same calibration dataset but with different methods or settings. The meta-model then learns to combine the outputs of base models in an optimal way to produce the final prediction. In this study, we utilized stacking generalization technique to combine the outputs of the DC-CARS-OLS model and SG-CARS-OLS model, aiming to leverage their respective strengths and enhance the final predictive capability and generalization performance of the regression model. The specific structure and computational flow of the stacking generalization model utilized in this study can be found in **Figure 5**, providing a visual representation of how the stacking generalization process is implemented.

The performance of the base models as well as the stacking generalization model is presented in **Table 8**. The performance of the stacking generalization model on the calibration set showed a decrease compared to the DC-CARS-OLS model. Besides, its performance has improved compared to the SG-CARS-OLS model on both the calibration and validation sets. These findings suggest that the stacking generalization model effectively addresses the overfitting issue observed in the DC-CARS-OLS model and further enhances the model’s fitting ability based on the SG-CARS-OLS model. By combining the strengths of both base models, the stacking technique successfully achieves improved overall performance and enhanced generalization ability.

**Table 8:** Regression results of the base models as well as the stacking generalization model.

Regression model	Calibration (Cross-Validation)			Validation		
	*RMSE_c_*	*MAPE_c_*	*RPD_c_*	*RMSE_p_*	*MAPE_p_*	*RPD_p_*
DC-CARS-OLS	**0.760**	0.047	1.372	1.189	0.077	0.844
SG-CARS-OLS	0.895	0.053	1.139	0.740	0.046	1.358
Stacking Generalization	0.782	**0.047**	**1.331**	**0.721**	**0.046**	**1.394**

The best scores are highlighted in bold.

**Figure 6:**
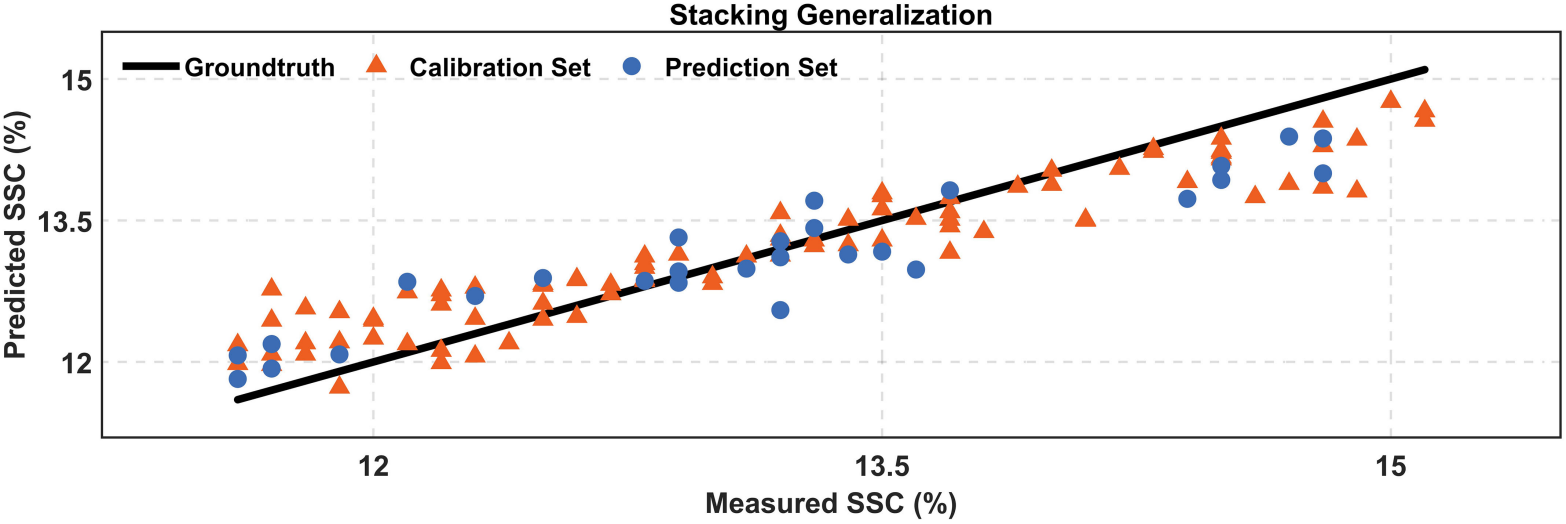
Comparison of the measured and predicted SSC.

The comparison between the experimentally measured and stacking generalization model-predicted values of SSC is shown in **Figure 6**. The close alignment of predicted SSC distributions across both calibration and prediction datasets underscores the model’s robustness, reflecting its capability to generalize well without succumbing to overfitting within the calibration phase.

This study’s approach is benchmarked against established methods in the field, with comparative results detailed in **Table 9**. Moen et al. (Moen et al., 2021) explored the link between kiwifruit spectral data and SSC using various machine learning approaches and determined that the optimal prediction was achieved by UVE-PLS model, yielding an $RMSE_{p}$ of 1.047. Zhou et al. (Zhou, 2022) also investigated this relationship and discovered that SVR model offered the best predictive accuracy with an $RMSE_{p}$ of 1.309. Meanwhile, Benelli et al. (Benelli et al., 2022) applied a PLS model leveraging hyperspectral imaging to assess kiwifruit maturity, attaining $RMSE_{c}$ and $RMSE_{p}$ values of 0.81 and 0.73, respectively. In our research, cross-validation within the calibration set was utilized to robustly detect overfitting, resulting in the most accurate predictions characterized by the lowest $RMSE_{p}$ in the validation set.

**Table 9:** Comparison of the prediction results with the other methods.

Literature	Method	Calibration (Cross-Validation)	Validation
		*RMSE_c_*	*RMSE_p_*
Moen et al., 2021	UVE-PLS	/	1.047
Zhou, 2022	SVR	/	1.309
Benelli et al., 2022	PLS	0.810	0.730
This study	Stacking generalization	**0.782**	**0.721**

The best scores are highlighted in bold.

## 4 Conclusion

In conclusion, our investigation reveals that feature engineering, particularly the application of the CARS method for feature selection, significantly enhances SSC prediction accuracy in kiwifruit using hyperspectral imaging. Through rigorous comparative analysis, we established that the DC-CARS-OLS model delivers the most accurate results in calibration, while the SG-CARS-OLS model excels in validation scenarios. These outcomes specifically highlight the critical nature of spectral feature selection in constructing effective predictive models. Additionally, the introduction of the stacking generalization technique has proven instrumental in amalgamating the predictive strengths of individual models, thereby mitigating overfitting, and refining overall regression accuracy. Our findings not only bolster the methodological framework for non-destructive SSC estimation in kiwifruit but also suggest a template for broader application in agricultural quality assessment. The practical upshot of our study is a robust, non-invasive approach that promotes the kiwifruit industry’s capability to ensure product quality, optimize resource use, and minimize waste. Ultimately, this research underlines the transformative potential of targeted feature engineering and advanced ensemble techniques in enhancing the precision of agricultural produce quality prediction models.

## Data availability statement

The raw data supporting the conclusions of this article will be made available by the authors, without undue reservation.

## Author contributions

WX: Data curation, Formal analysis, Investigation, Methodology, Writing – original draft. LW: Conceptualization, Investigation, Writing – review & editing. WC: Data curation, Writing – review & editing. XY: Visualization, Writing – review & editing. YL: Supervision, Writing – review & editing.
